# Treatment of High Risk Sertoli–Leydig Cell Tumors of the Ovary Using a Gonadotropin Releasing Hormone (GnRH) Analog

**DOI:** 10.1002/pbc.24382

**Published:** 2012-11-28

**Authors:** Harsha Prasada Lashkari, Ruth Nash, Assunta Albanese, Bruce Okoye, Robert Millar, Kathy Pritchard-Jones

**Affiliations:** 1 The Royal Marsden Hospital NHS TrustSutton, Surrey, UK; 2 St. Georges Healthcare NHS TrustTooting, London, UK; 3 Mammal Research Institute, University of PretoriaSouth Africa; 4 UCT/MRC Receptor Biology Unit, University of Cape TownSouth Africa; 5 Centre for Integrative Physiology, University of EdinburghScotland, UK; 6 Institute of Child Health, University College LondonLondon, UK

**Keywords:** GnRH analog, leuprorelin, Sertoli–Leydig cell tumor (SLCT), sex cord stromal cell tumor

## Abstract

Sertoli–Leydig cell tumors are rare ovarian neoplasms. We report two unusual cases with bilateral SLCTs suggesting evidence of genetic predisposition and at high risk of recurrence. To reduce this risk, we exploited the use of GnRH analog to lower gondadotropin and potentially directly inhibit the tumors through expressed GnRH receptors. We used it as maintenance antitumor therapy for 2 years after completion of chemotherapy, to cover the period of risk for recurrence. Both patients remain in complete remission at >2 years after completing leuprorelin therapy. Of note, both patients carry DICER1 mutations, frequently found in pleuropulmonary blastoma syndrome. Pediatr Blood Cancer 2013; 60: E16–E18. © 2012 Wiley Periodicals, Inc.

## INTRODUCTION

Sertoli–Leydig cell tumor (SLCT) is a type of ovarian sex cord-stromal tumors 1 which occurs most frequently in the second and third decades. They are generally low-grade malignancies, and less than 20% become malignant 1, 2. We present two unusual high-risk cases where there was evidence of genetic predisposition (metachronous tumor discovered at relapse in one case, the other was a second primary tumor in a survivor of Wilms tumor). As both cases fell into a category of expected event free survival (EFS) of <50% 3, we wished to utilize maintenance antitumor therapy to cover the highest risk period of relapse 4. We chose a long acting GnRH analog (leuprorelin) to suppress any residual tumor cell proliferation.

## PATIENTS AND RESULTS

### Case 1

**Fig. 1:**
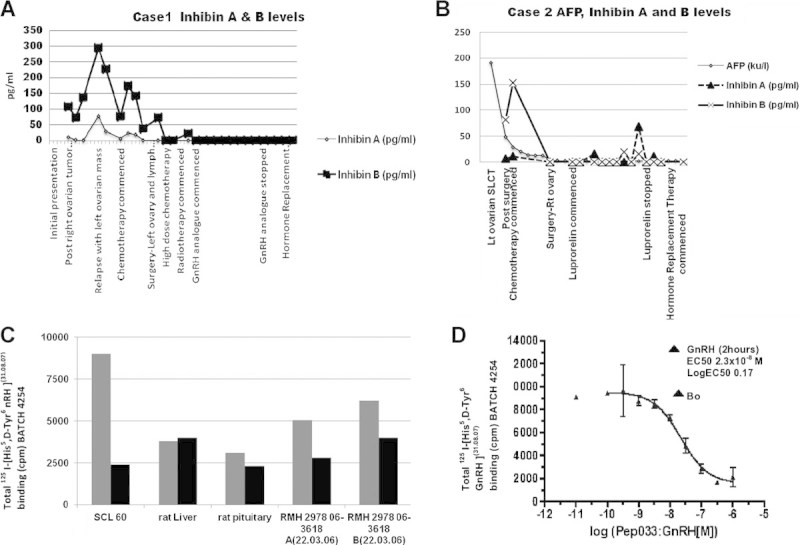
Case 1 inhibin A and B levels (**A**) and case 2 AFP, inhibin A and B levels (**B**) [normal range—inhibin A in premenopausal women varies with the cycle <7–69 pg/ml and inhibin B in girls aged 11–12 years as per Women and Infants Hospital, Rhode Island, US is —<10–186 pg/mol. Alfa-fetoprotein (AFP) is <10 ku/L]. GnRH receptor study on the case 1 specimen (**C**) [cell line SCL 60 expressing GnRH receptor, pituitary membranes as positive control and liver membranes as a negative control. The second column of each is the non-specific binding (10^−6^ M ligand). Both tumor specimen A and B had specific binding]. Dose–response binding curve of cell membranes of case 1 specimen demonstrating high affinity binding sites suggestive of GnRH receptors (**D**).

A 12-year old female was diagnosed with right ovarian mass after presentation with a 7-month history of abdominal distension. Tumor markers were normal except for moderately raised AFP (Alfa fetoprotein-35 ku/L) and inhibin A and B (10.2 and 108.0 pg/ml, respectively (Fig. 1A) measured postoperatively). The mass was completely resected and histopathology revealed SLCT stage 1A.

Fourteen months later, a left ovarian and a right para-aortic lymph node mass were detected on routine ultrasound scan. Tumor markers were normal except for raised inhibin B 294 pg/ml (normal range <8–196 pg/ml, Fig. 1A). The lymph node biopsy confirmed the recurrence of SLCT. Chemotherapy consisting of cisplatin, ifosfamide, and etoposide (PIE) was administered as per the schedule used by the German Pediatric Oncology Group for germ cell tumor 5.

**Fig. 2:**
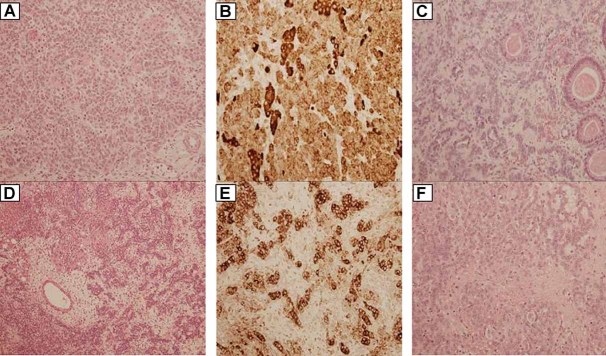
Case 1 left side tumor [H&E 200×] showing solid nests, cords of Sertoli cells with clusters of Leydig cells (**A**) and its inhibin staining (**B**) [inhibin 400×]. Case1 para aortic lymph node [H&E 200×] deposit [14 months later] predominant Sertoli cell features, with small tubules and cords in a hyalinised background and occasional incorporated larger “heterologous” glandular elements with retiform areas (**C**) [consistent with metastasis from the original right ovarian tumor that had shown similar features]. Case 2 left side tumor [H&E 100×] cells with a partly retiform arrangement in a loose stroma and intermingled clusters of Leydig cells (**D**) and its inhibin staining (**E**) [inhibin 200×]. Case 2 right side tumor [H&E 200×]-loose sheets of Sertoli cells and sparse interspersed Leydig cells (**F**).

Following four courses of chemotherapy, the para-aortic lymph node and left ovary were resected. Microscopically, the lymph node metastases showed predominant Sertoli cell features, consistent with metastasis from the original right ovarian tumor (Fig. 2C). By contrast, the left ovarian tumor showed groups of definite Leydig cells (Fig. 2A and B**)** with a sheet like structure and was very different histologically from the original right-sided ovarian tumor. For this reason, the left ovarian tumor was felt to represent a metachronous contra lateral new primary tumor.

Due to the poor survival despite intensive therapy reported in the German series 4, we consolidated this remission with high dose therapy (carboplatin, etoposide, and melphalan) with autologous stem cell rescue and radiotherapy (54 Gy) to the para-aortic lymph nodal area. On recovery, she commenced the GnRH analog leuprorelin acetate for 2 years (leuprorelin 3.75 mg, intramuscular (IM), three weekly for three cycles, followed by a longer acting form (11.25 mg IM injection every 9 weeks). Efficacy of suppression of LH/FSH was monitored regularly. The treatment was well tolerated. Six months after stopping GnRH analogs, hormonal replacement therapy with estradiol was introduced. She remains well 6 years from time of relapse and 3 years from end of leuprorelin treatment.

### Case 2

A 12-year old female diagnosed with large left ovarian mass after presentation with history of menorrhagia and of an abdominal mass. She was previously treated at the age of 8 years for a Wilms tumor. She also has cystic multinodular goiter. Further investigations showed no evidence of metastases. Preoperative AFP was raised (191 ku/L) and inhibin B was in the higher range (81.4 pg/ml) when first measured postoperatively.

She proceeded to complete resection of the left ovary and complete macroscopic excision of a small nodule noted on the right ovary during the surgery. Histopathology (Fig. 2D and E) of both tumor and the nodule showed intermediate to poorly differentiated SLCT (stage 1C).

Postoperative MRI showed a right ovarian mass. Following two courses of PIE chemotherapy, the right ovary was completely removed and histopathology (Fig. 2F) showed a SLCT with intermediate differentiation, stage 1C. She commenced leuprorelin acetate according to the same schedule as case 1 after completing four courses of chemotherapy. The interval between injections was reduced to 6 weeks in order to keep LH and FSH levels fully suppressed. She completed 2 years of this therapy and hormone replacement therapy was commenced similar to case 1. She remains in complete remission at 4 years from diagnosis and 30 months after completion of GnRH therapy. The one abnormal inhibin A level measured during follow up was ascribed to a laboratory error and subsequent levels were normal (Fig. 1B).

### Detection of GnRH Receptors

The receptor binding studies were conducted on frozen tumor from case 1 6. Membranes from a HEK293 cell line stably expressing the GnRH receptor (SCL60) and rat pituitary were used as positive controls and liver membranes as negative control. Two separate samples of Case 1 tumor showed specific binding of radiolabelled GnRH analog as did SCL60 and pituitary (Fig. 1C and D).

## DISCUSSION

There are few data in the literature on risk-adapted therapeutic strategies in ovarian sex cord stromal cell tumors in children. Schneider et al. 4 described 54 cases documented prospectively and defined risk groups with respect to stage; completeness of resection; histologic appearance, such as differentiation and proliferative activity of the tumor. Surgery remains the mainstay of initial management. Cisplatin based chemotherapy constitutes an important role as adjuvant therapy for those patients with SLCT stage I, poorly differentiated and those containing heterologous elements.

In 1975, Stadel 7 proposed the “gonadotropin theory,” hypothesizing that exposure to high gonadotropin levels favor malignant transformation. SLCTs express receptors for follicle stimulating hormone (FSH) which has been shown to support the growth of granulosa cell tumors in nude mice 8. GnRH analog therapy at high doses can act to suppress the receptor activity and has been suggested as treatment for progressive ovarian tumors that have failed to respond to chemoradiation 9. The apparent efficacy of GnRH analog in our SLCT patients which lowered gonadotropins may support this theory. There may have also been direct antiproliferative effects as described in a number of reproductive tissue cancers, including ovarian cancer 10–15. Although there are case reports about the use of GnRH analog for SLCTs of the ovary in adults 16, no data have been reported in children. Whilst we could not demonstrate tumor response in the absence of measurable disease or raised tumor markers, we are encouraged by the duration of clinical remission in both patients and their lack of problems on introduction of hormone replacement therapy.

Recent advances in the understanding of the genetics of SLCTs have provided insight into the unusual clinical phenotype in the two cases presented here. Both were included in a study of the rare pleuropulmonary blastoma syndrome of which SLCTs have recently been shown to form part of the spectrum 17. This syndrome is due to constitutional heterozygous mutations in the DICER1 gene, the master regulator of micro RNA production. Both cases presented had constitutional DICER1 mutations. DICER1 mutations can cause a range of phenotypes from asymptomatic to various tumors such as cystic nephroma, pleuropulmonary blastoma, thyroid cysts, SLCTs, and Wilms tumor. Case 2 also has thyroid cysts, now recognized as part of the DICER1 syndrome 17.

In conclusion, the prolonged remission in two cases presented here suggests that GnRH analogs may have a therapeutic role in high risk SLCTs. While we do not have direct evidence for the efficacy of GnRH analogs with potential influence of high dose therapy and stem cell rescue on case 1, our findings set the scene for further studies for maintenance therapy using GnRH analogs in SLCTs.
